# Incidence of Invasive Fungal Infections in Liver Transplant Recipients under Targeted Echinocandin Prophylaxis

**DOI:** 10.3390/jcm12041520

**Published:** 2023-02-14

**Authors:** Robert Breitkopf, Benedikt Treml, Katharina Simmet, Zoran Bukumirić, Margot Fodor, Thomas Senoner, Sasa Rajsic

**Affiliations:** 1 Department of Anesthesiology and Intensive Care Medicine, Medical University Innsbruck, 6020 Innsbruck, Austria; 2 Institute of Medical Statistics and Informatics, Faculty of Medicine, University of Belgrade, 11000 Belgrade, Serbia; 3 Department of Visceral, Transplantation and Thoracic Surgery, Medical University of Innsbruck, 6020 Innsbruck, Austria

**Keywords:** antimycotic, antifungal, adverse events, complications, fungi, invasive infection, liver, mortality, prophylaxis, transplantation

## Abstract

Invasive fungal infections (IFIs) are one of the most important infectious complications after liver transplantation, determining morbidity and mortality. Antimycotic prophylaxis may impede IFI, but a consensus on indication, agent, or duration is still missing. Therefore, this study aimed to investigate the incidence of IFIs under targeted echinocandin antimycotic prophylaxis in adult high-risk liver transplant recipients. We retrospectively reviewed all patients undergoing a deceased donor liver transplantation at the Medical University of Innsbruck in the period from 2017 to 2020. Of 299 patients, 224 met the inclusion criteria. We defined patients as being at high risk for IFI if they had two or more prespecified risk factors and these patients received prophylaxis. In total, 85% (190/224) of the patients were correctly classified according to the developed algorithm, being able to predict an IFI with a sensitivity of 89%. Although 83% (90/109) so defined high-risk recipients received echinocandin prophylaxis, 21% (23/109) still developed an IFI. The multivariate analysis identified the age of the recipient (hazard ratio—HR = 0.97, *p* = 0.027), split liver transplantation (HR = 5.18, *p* = 0.014), massive intraoperative blood transfusion (HR = 24.08, *p* = 0.004), donor-derived infection (HR = 9.70, *p* < 0.001), and relaparotomy (HR = 4.62, *p* = 0.003) as variables with increased hazard ratios for an IFI within 90 days. The fungal colonization at baseline, high-urgency transplantation, posttransplant dialysis, bile leak, and early transplantation showed significance only in a univariate model. Notably, 57% (12/21) of the invasive Candida infections were caused by a non-albicans species, entailing a markedly reduced one-year survival. The attributable 90-day mortality rate of an IFI after a liver transplant was 53% (9/17). None of the patients with invasive aspergillosis survived. Despite targeted echinocandin prophylaxis, there is still a notable risk for IFI. Consequently, the prophylactic use of echinocandins must be critically questioned regarding the high rate of breakthrough infections, the increased occurrence of fluconazole-resistant pathogens, and the higher mortality rate in non-albicans Candida species. Adherence to the internal prophylaxis algorithms is of immense importance, bearing in mind the high IFI rates in case algorithms are not followed.

## 1. Introduction

Liver transplantation presents a standard of care and life-saving procedure for end-stage liver diseases. With the constant development of surgical techniques, complications’ management, and the use of the newest immunosuppressive regimens, the one-year mortality rate dropped to 10%, enabling a 10-year survival rate of more than 60% [1,2,3,4,5,6].

Recent retrospective studies on more than 50 000 liver transplant recipients associated the early postoperative period with a higher mortality risk (3.5% within the first month after liver transplantation), with infections being the most common cause of death (39%) [5,7]. More than 50% of deaths within the first week after transplant, excluding the first 48 postoperative hours, are attributable to cardiovascular, cerebrovascular, pulmonary, or hemorrhagic events, with infections (13%) being the second most common reason for early mortality. Thereafter, death became predominantly associated with infections [5,8,9]. 

Liver transplant recipients are prone to bacterial infections related not only to abdominal collections, catheters, or the biliary tree but also to opportunistic infections, including invasive fungal infections (IFIs), and multidrug-resistant organisms due to iatrogenic immunosuppression [10,11,12,13]. Most infections are caused by bacteria (70%), followed by viruses and fungi [14]. 

Invasive fungal infections are one of the most important infectious complications after liver transplantation, determining its morbidity and mortality [15,16,17,18]. Historically, the incidence of IFI in liver transplant recipients has been reported between 5% and 42%, with an associated mortality rate of 25% to 71% [19,20,21]. In the case of invasive aspergillosis (IA), this may be high as 80% [22,23]

Established risk factors for IFI include postoperative renal replacement therapy, cytomegalovirus (CMV) infection, re-transplantation, perioperative fungal colonization, massive blood transfusion (of ≥40 units of packed red blood cells), Roux-en-y choledochojejunostomy, re-operation after transplantation, pretransplant serum creatinine (SCr) of more than 3 mg/dL, operative time longer than 11 h, antibiotic pretreatment, postoperative bile leakage, and an elevated MELD score [18,24,25,26,27,28]. 

Given the incidence and outcome determining effects of IFI, the question of an indication for antimycotic prophylaxis arises. Earlier studies reported a reduced incidence of infections in the case of antimycotic prophylaxis utilization but without impact on the overall mortality [26,29,30,31,32,33,34]. Considering the potential for the emergence of resistance, universal prophylaxis was discouraged. Instead, targeted prophylaxis has been recommended, selectively directed to high-risk patients only [35]. 

Nevertheless, a consensus on defining high-risk patients, the agent, or the duration of antimycotic prophylaxis for liver transplant recipients is still missing. Therefore, we aimed to investigate the incidence of IFI under targeted echinocandin prophylaxis in predefined adult high-risk first-time orthotopic liver transplant (OLT) recipients. Moreover, we reassessed previously reported predictors and risk factors and compared the demographic and clinical characteristics of a unique population of critically ill patients while focusing on adverse events and outcomes.

## 2. Materials and Methods

### 2.1. Study Design and Population

We retrospectively reviewed the electronic medical charts of all patients undergoing orthotopic, first-time liver transplantation between January 2017 and December 2020 at the Medical University of Innsbruck, Department of Anesthesiology and Intensive Care Medicine. The primary endpoint was the occurrence of an early postoperative proven or probable IFI within 90 days after transplantation. The perioperative care of patients was conducted at a tertiary intensive care unit (ICU) specialized for transplanted patients. 

We included all adult patients receiving OLT or combined liver−kidney transplantation. Excluded were patients younger than 18 years, living donor liver recipients, re-transplantations occurring more than 90 days after the initial operation, and multivisceral transplantations. In the case of liver re-transplantation within 90 days after the first transplant, only data from the first operation were analyzed. 

This retrospective study was approved by the Ethics Committee of the Medical University of Innsbruck, Austria (Number 1126/2022).

We prepared and revised our work according to the strengthening of the reporting of observational studies in epidemiology (STROBE) statement—a checklist of items (Table S1). 

### 2.2. Definition of an Invasive Fungal Infection (IFI)

The diagnosis of a “proven” IFI was based upon the most recent definitions of the IFI from the European Organization for Research and Treatment of Cancer/Invasive Fungal Infections Cooperative Group and the National Institute of Allergy and Infectious Diseases Mycoses Study Group (EORTC/MSG) Consensus Group [36]. Depending on the level of probability for IFI diagnosis in a critical care setting, a “probable” disease was diagnosed upon the recommendations of the EORTC/MSGERC ICU Working Group [37].

Criteria for a “proven” IFI require either a blood culture or microscopic (direct microscopic, histopathologic, or cytopathologic) analysis of a specimen, which was obtained under sterile conditions (including a freshly placed (<24 h) drain) from a site showing clinical or radiological signs of infection. Candidemia was defined as the isolation of *Candida* spp. from at least one blood culture.

The diagnosis of a “probable” invasive candidiasis (IC) was based on the presence of at least one clinical criterion (hepatosplenic lesions by computed tomography, compatible ocular findings by fundoscopic examination, radiological or clinical (non-pulmonary) abnormalities corresponding to infectious disease process being otherwise unexplained) in combination with at least one mycological criterion (i.e., finding of candida in an intra-abdominal specimen obtained surgically or within one day from external drainage or positive serum 1.3-β-d-glucan in two consecutive samples). A non-candidemia intra-abdominal candidiasis (IAC) was rated as invasive only in cases with histopathologic or direct microscopic examination of perioperatively sampled sterile fluid or tissue. 

Positive samples taken via drains >24 h after surgery as well as candida isolation from respiratory secretions, stool, skin, wound sites, and an asymptomatic candiduria, were interpreted as colonization or, in the case of clinical signs of sepsis, as “possible” infection [37,38,39,40,41]. 

A “probable” IA was diagnosed after recovery of *Aspergillus* spp. either via cytology, direct microscopy, and/or culture in a lower respiratory tract specimen, or via a galactomannan antigen index >0.5 in plasma/serum, and/or galactomannan antigen >0.8 in bronchoalveolar lavage fluid (BALF) in case of at least one radiological CT sign for a lower respiratory tract fungal disease (wedge-shaped and segmental or lobar consolidation; cavity; dense, well-circumscribed lesions with or without a halo sign, air crescent sign) or the bronchoscopic proof of a tracheobronchitis (tracheobronchial ulceration, pseudomembrane, nodule, plaque, or eschar) consistent with an otherwise unexplained pulmonary infectious-disease process.

In the case of an IFI, computer tomography, transoesophageal echocardiography, and fundoscopy were routinely performed to detect organ involvement.

### 2.3. Immunosuppressive Regimen and Prophylaxis

The local standard algorithm for immunosuppressive therapy in adults, ABO-compatible OLT, comprised an intraoperative steroid bolus of 500 mg methylprednisolone and subsequent tapering over five weeks (except for patients with autoimmune diseases) in a triple combination with a calcineurin inhibitor (CNI) and an antimetabolite. The non-depleting interleukin 2 receptor antagonist (IL-2Ra, basiliximab) was used to delay the CNI introduction as part of a renal−sparing strategy or in the case of a combined kidney−liver transplantation.

A once-daily, prolonged-release tacrolimus (PR-Tac) was used as the first-line CNI. In case of Tac-related side effects (e.g., long-QT syndrome, tremor, vomiting, alopecia, diarrhea, headache, or dyspepsia) it was switched to cyclosporine A (CsA). Target C0 levels of Tac and CsA were 7–10 and 150–200 ng/mL in the first three months, respectively. Mycophenolate mofetil (MMF) was used as a first-line antimetabolic agent. In case of gastrointestinal side effects, enteric-coated mycophenolate sodium (EC-MPS) or azathioprine (AZT) were used instead. 

Elective recipients received preoperative selective digestive decontamination with oral amphotericin B and nonabsorbable antibiotics, followed by an extended-spectrum perioperative antibacterial prophylaxis with piperacillin-tazobactam for five days; levofloxacin was used alternatively for patients allergic to β-lactam agents.

Patients at high risk of CMV infection, hence CMV seronegative recipients from seropositive donors, received valganciclovir prophylaxis for 3–6 months. A preemptive approach based on weekly polymerase chain reaction (PCR) surveillance testing was followed in all other patients. Antiviral therapy was then initiated after the detection of CMV viremia but prior to the onset of clinical symptoms. 

Routine microbiological screening was performed on all patients at least once per week during the ICU stay or earlier if indicated by a critical care specialist. 

According to our protocol, targeted antimycotic prophylaxis was performed in high-risk liver transplant recipients defined by 2 or more of the 15 perioperative risk factors (Table 1).

In the case of an uncomplicated postoperative course, the prophylaxis was carried out using echinocandins (micafungin, anidulafungin) over a period of 7–14 days. We initially used micafungin until the end of March 2019, followed by anidulafungin from then on. The decision to exchange the echinocandin was based on an overarching recommendation of the local drug commission. Depending on the clinical course and the occurrence of additional postoperative risk factors, prophylaxis would be prolonged for up to 28 days, based on the decision of the attending physician. Reasons for an earlier termination were the completion of prophylaxis at discharge or missing clinical signs of infection, death, or switch to a therapeutic regime in case of diagnosed infection.

In case of pre-existing fungal colonization with echinocandin-resistant *Candida* spp. (e.g., *Candida parapsilosis*) or *Aspergillus* spp., the prophylaxis was switched to fluconazole, voriconazole, or liposomal amphotericin B.

The following antifungal dosages were considered adequate: micafungin (100 mg/d); anidulafungin (200 mg loading dose, followed by 100 mg/d); fluconazole (800 mg loading dose, 1200–1600 mg in case of a body mass index (BMI) above 30), followed by a daily dosage of at least 400 mg (600–800 mg, BMI > 30); voriconazole (2 loading doses of 6 mg/kg, every 12 h), with a maintenance dose (4 mg/kg twice a day) and later on adjusted according to a weekly performed therapeutic drug monitoring; and liposomal amphotericin B (L-AmB, 3 mg/kg per day). These dosages refer to patients with normal hepatic and renal function; otherwise, the dose was adjusted accordingly.

### 2.4. Surgical Technique

A standard OLT was defined as deceased donor transplantation of a standard criteria donor (SCD) whole organ after static cold storage (SCS) from a donation after brain death (DBD). The recipient hepatectomy was performed by retrohepatic caval resection without a veno-venous bypass, and the biliary anastomosis by duct-to-duct reconstruction. Deviations from this technique (e.g., split liver donation, extended criteria donation (ECD), donation after circulatory determination of death (DCD), the use of a veno-venous bypass, an inferior vena cava preservation by “piggyback” technique, or a Roux-en-y choledochojejunostomy) were recorded.

Extended criteria donors were defined according to the Eurotransplant Foundation rules by the following criteria: donor age >65 years, ICU stay with ventilation > 7 days, donor BMI > 30 kg/m^2^, hepatic steatosis > 40%, serum sodium > 165 mmol/L, alanine aminotransferase (ALT) > 105 U/L, aspartate aminotransferase (AST) > 90 U/L, total bilirubin > 3 mg/dL, and DCD [42].

Normothermic machine perfusion (NMP) was introduced at our center in February 2018 and has meanwhile been implemented in a daily routine for the following indications [43]:

- Donor-related: in cases of ECD, especially if prolonged ischemia times are expected
- Recipient-related: in cases of surgically highly complex recipients or high-risk patients
- Logistic-related: in case of limited resources (e.g., parallel organ transplantations or overlap with other urgent interventions).

### 2.5. Data Acquisition

We obtained the data on (1) sociodemographic characteristics of the transplant recipients, their comorbidities (Charlson Comorbidity Index), the severity of disease (MELD score, SAPS III score), and the underlying indication for OLT, (2) basic data on organ donation and preservation, surgical implantation technique and procedure characteristics; (3) information on immunosuppression and prophylaxis-related adverse events, postoperative complications, ICU- and hospital stay, and patient survival. 

The incidence of mycotic colonization, local and systemic infection of mycotic origin, date and causes of death, and potential risk factors for IFI were further collected. Death was attributed to mycotic infection if there was an ongoing positive culture or infectious process at the death occurrence. Compliance with the algorithm was defined by the use of the antifungal drug. 

Microbiological data were recorded throughout the 90 days from the transplantation. All positive microbiological findings were screened for contamination. The organ and patient follow-up in regard to survival was limited to one year. Two authors independently evaluated each medical chart and extracted the data in a predesigned case report form.

### 2.6. Outcomes

The aim of our work was to evaluate the feasibility and efficacy of targeted antifungal echinocandin prophylaxis among OLT recipients. 

The primary endpoints were the incidence of an IFI among high-risk OLT recipients within 90 days of transplantation, under targeted echinocandin prophylaxis, and compliance with the risk stratification algorithm. The secondary endpoints included the evaluation of risk factors, the spectrum of fungal pathogens, the attributable 90-day mortality for a diagnosed IFI, one-year patient survival, postoperative course, and complications (e.g., ICU length of stay, the incidence of postoperative renal failure, ICU readmission, and re-operation rates), and finally, possible adverse events of the antifungal prophylaxis.

### 2.7. Statistical Analyses

Statistical analyses were performed using the SPSS (Version 22.0. Released 2013, Armonk, NY, USA: IBM Corp.). All statistical assessments were two-sided, and a significance level of 0.05 was applied. Depending on the type of variables and normality of the data distribution, results are presented as frequency (percent), median (range, minimum–maximum), and mean with standard deviation. For parametric data, the independent samples *t*-test was used, and the Mann–Whitney U test for ordinal and numeric data with non-normal distribution. Fisher’s exact test and Chi-square test were used to test differences between the nominal data (frequencies). Potential risk factors for IFI occurrence were analyzed in a univariate Cox proportional hazards model. Covariates with a significance level of *p* < 0.05 were included in a multivariate model. 

## 3. Results

### 3.1. Demographic and Clinical Characteristics

**Figure 1:**
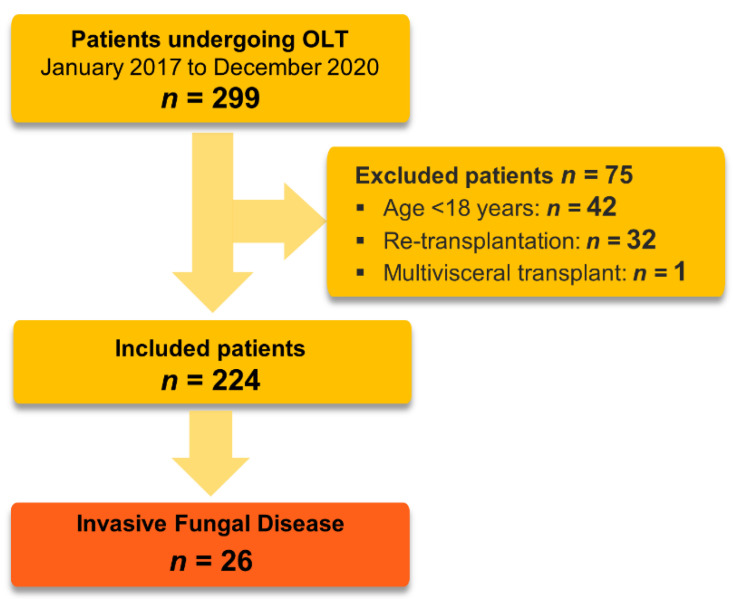
Flowchart of patient selection. OLT: orthotopic liver transplantation.

During the observation period, 299 patients underwent OLT, 289 (97%) isolated liver transplantation, and 10 (3%) combined liver−kidney transplantation. In total, 224 patients met the inclusion criteria Figure 1.

The mean age of the analyzed population was 57 ± 11 years, with 77% being males (*n* = 172). Included patients had a median Charlson comorbidity index of 4 (0–12), a MELD score of 14 (6–40), and a mean SAPS III score of 45 ± 9. The baseline demographic and clinical characteristics are summarized in Table 2.

The main indication for OLT was malignancy and other tumors (92, 41%), followed by alcoholic liver disease (56, 25%), cholestatic liver disease (17, 8%), and non-alcoholic fatty liver disease (14, 6%), Table 2. High urgent OLT due to acute liver failure was performed in 5% of the cases. 

A total of 79% of the deceased donor donations were made under ECD, with 9% as DCD and 6% as split-liver transplantations. In 68%, organ preservation was done by SCS, and NMP was used in 32% (72) of cases. Caval replacement was performed in 214 (97%) cases, while the piggyback technique was performed in 7 cases (3%). Duct-to-duct anastomosis was initially performed in most of the patients (209, 93%), Roux-en-Y choledochojejunostomy in 7% as the primary biliary reconstruction during OLT, and in another 4% to treat biliary complications.

The immunosuppression was carried out with high-dose prednisone induction, followed by tacrolimus, MMF, and low-dose prednisone in 87% (195/224) of the cases; the low-dose prednisone was tapered down to 20 mg per day within 20 days. In 17 cases, tacrolimus was replaced by cyclosporine A and in the 10 cases of combined kidney−liver transplantation, basiliximab induction was additionally combined.

### 3.2. Risk Factors for Infection

Post-transplantation dialysis was the most common risk factor (99, 44%), followed by relaparotomy (77, 34%), CMV viremia (64, 29%), and bile leak (14, 15%), see Table 3.

The univariate Cox regression analyses identified the age of the recipient, fungal colonization at baseline, high-urgency or split liver transplantation, intraoperative blood transfusion of more than 40 PRBC, bile leak, relaparotomy, early re-transplantation, donor-derived infection, and posttransplant dialysis as independent risk factors for IFI development (Table 4). Finally, recipient age, split liver transplantation, intraoperative blood transfusion of more than 40 PRBC, donor-derived infection, and relaparotomy had an increased hazard ratio for IFI within 90 days in the multivariate Cox regression model (Table 4).

Five of the seven patients (71%) with donor-derived infection developed an IFI, mainly caused by *Candida albicans* (60%, 3/5). Three patients died after the end of the 90-day observation period but within the first year after OLT. The other two patients developed a graft failure, of which one needed an early, high-urgent re-transplantation.

Of the 224 patients, 109 (49%) patients were consequently classified as high-risk for IFI (according to the applied risk stratification algorithm), and 115 (51%) had a low-risk profile. All OLT recipients were solid organ transplant recipients with impaired cutaneous barriers to bloodstream infections (e.g., presence of indwelling vascular access devices) and impaired gut wall integrity after recent abdominal surgery. 

### 3.3. Targeted Antimycotic Prophylaxis

**Figure 2:**
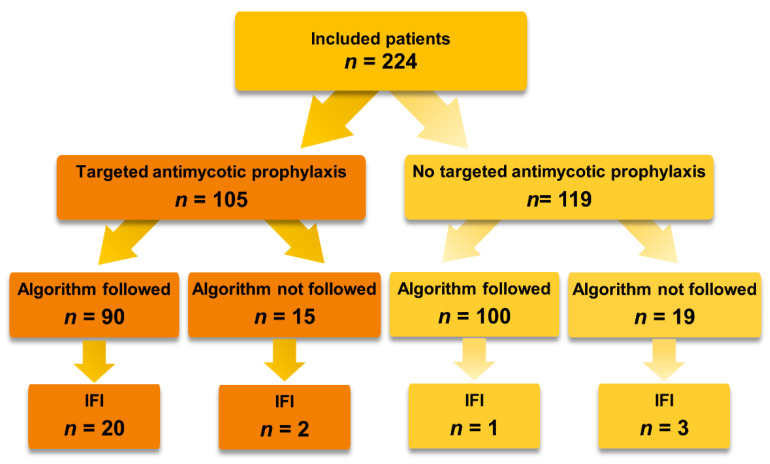
Flowchart of Targeted Antimycotic Prophylaxis (TAP) adherence.

Out of all included patients, 190 (85%) were treated according to the prophylaxis algorithm and 90 (47%) patients received antifungal prophylaxis, Table S2. Fifteen patients received prophylactic treatment with less than two risk factors (Figure 2). In 7 out of 15 cases, the empirical decision was based purely on the clinical assessment of an initially suspected septic condition by the critical care specialist; in 8 cases, antimycotic prophylaxis or systematic treatment of a non-invasive infection was indicated for other reasons (earlier IC (*n* = 1), thrush esophagitis (*n* = 2), HIV infection (*n* = 1), acute cellular rejection (*n* = 2), pre-existing severe COPD (*n* = 1), and an initially suspected but later on falsified donor-derived infection (*n* = 1)). Contrary to the usual procedural routine, 19 patients received no prophylaxis despite an increased risk profile due to situational violations (*n* = 15) in transplantations outside of routine working hours (night work, weekends) and as a result of capacity restrictions during the COVID-19 pandemic (*n* = 4).

The used substances were initially micafungin (67/105, 64%) and anidulafungin (33/105, 31%). Five patients (5%) were treated differently due to specific clinical reasons: one patient was already on antifungal treatment with voriconazole prior to being called for transplantation; two were treated with voriconazole as pulmonary aspergillosis was suspected; in two patients, the prophylaxis was adapted to fluconazole and voriconazole due to a donor-derived infection (by a proven *Geotrichum capitatum* in the second case by an initially suspected and later falsified *Aspergillus* spp.).

The overall median duration of the antimycotic prophylaxis was 9 (1–49) days. Within the IFI patients, the median duration of prophylaxis up to the diagnosis was 8.5 (1–40) days. The duration of prophylaxis was rated as one day in the cases where the start of prophylaxis was initially indicated by the mentioned criteria, but a diagnosis of a proven or probable IFI was made already during the same day, when the newest findings were available, indicating the need for therapeutic treatment.

In 3 patients out of 22 (14%), patients with prophylaxis IFI occurred after a 2–3 week lasting prophylaxis (14, 17, and 20 days). Finally, in 19 (86%) cases, IFI occurred during the ongoing exposure to antifungal drugs, from which 11 met the high-risk criteria and were treated with antifungal drugs from the day of transplantation. Further, 8 patients (36%) met the criteria of the algorithm only in the postoperative course, and the prophylaxis was started with a median delay of 15 (11–55) days: 4 because of a bile leak, 2 because of an otherwise caused re-operation, and 2 in consequence of an endoscopic biliary intervention. 

### 3.4. Incidence of Invasive Fungal Infections

Invasive fungal infection developed in 26 (12%) patients. The incidence ranged from 4% to 20% in the years studied, with the highest number of transplants occurring in 2018 (*n* = 70) but the highest incidence of IFI (20%) in 2020, Table 5.

Twenty-three IFIs occurred within the high-risk group and three within the low-risk group. Consequently, 198 patients developed no IFI, 86 within the high-risk group, and 112 within the low-risk group. This leads to an algorithm sensitivity of 89% (95%CI, 69.9–97.6), and specificity of 57% (95%CI, 49.4–63.6), with a negative predictive value of 97% (95%CI, 92.6–99.5) and positive predictive value of 21% (95%CI, 13.9–30.0).

Twenty-one patients developed a proven or probable IC (Table 6), and twenty-five (11%) had a superficial Candida infection, including one patient with biopsy-proven esophagitis.

Five patients (2%) developed IA with *Aspergillus fumigatus*, four of them occurred in combination with another IFI, and none of them survived to discharge, Table 7. 

Two patients (1%) developed another form of an IFI. In one patient with clinical and radiological abnormalities consistent with an otherwise unexplained infectious-disease process, the DNA of Saccharomyces spp. was detected more than once by real-time PCR in a surgically obtained intra-abdominal specimen. 

In the second case, a recipient sustained an invasive *Geotrichum capitatum* infection transmitted by organ donation. The infection was diagnosed by several blood cultures yielding *Geotrichum capitatum* as well as its recovery in several surgically obtained intra-abdominal specimens and in drainage fluid of freshly placed drains. Moreover, fungal endophthalmitis was diagnosed by fundoscopy and later confirmed by culture and PCR in a biopsy.

### 3.5. Composition and Sites of Pathogens

**Figure 3:**
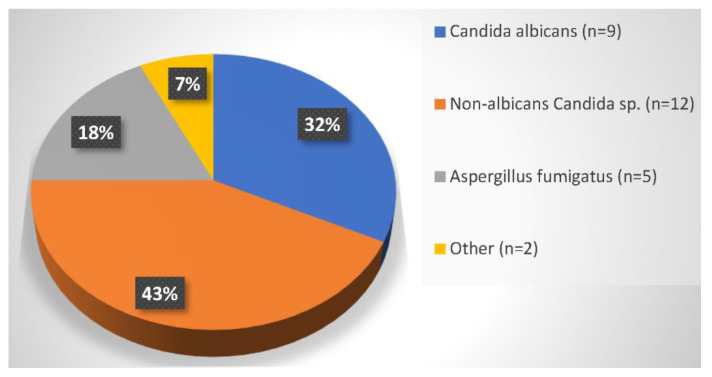
Composition of pathogens.

The identified IFI pathogens were *Candida* (21, 81%), *Aspergillus* (5, 19%), and once each *Saccharomyces* spp. and *Geotrichum capitatum*. Two times a non-albicans infection went along with an IA, see Figure 3. *Mucor circinelloides, Fusarium* spp., and *Penicillium* spp. were each identified once as fungal co-infection pathogens.

*Candida albicans* accounted for 43% (9/21 patients) of the invasive and 52% (13/25) of the superficial Candida infections. The most often localization was in abdominal specimens (8/9, 89%), blood (2/9, 22%), and one each with primary and secondary candidemia. 

Among the non-albicans Candida species being isolated from invasive infections, *C. glabrata* and *C. dubliensis* predominated (each 4/12, 33%), followed by *C. krusei* (3/12, 25%) and *C. parapsilosis* (1/12, 8%). Non-albicans Candida species were mostly identified from abdominal specimens (10/12, 83%), four of these with secondary candidemia (4/12, 33%), and two catheter-related (2/12, 17%). Non-albicans Candida species were further identified from the blood (6/12, 50%), in two cases as primary candidemia and one as catheter-related. 

All mold infections (including *Aspergillus* spp., as well as the non-Aspergillus molds *Mucor circinelloides, Fusarium* spp., and *Penicillium* spp.) were isolated from respiratory specimens. The one invasive *Geotrichum capitatum* infection presented clinically with fungemia, peritonitis, and endophthalmitis, and the invasive Saccharomyces infection with peritonitis. 

### 3.6. Time to Diagnosis

The median time to diagnosis was 13 days (3–77) for IC and 36 days (9–78) for invasive pulmonary aspergillosis. In 14 (54%) cases, the diagnosis occurred within two weeks after transplantation, and in 18 (69%), within one month. Candida species constituted 83% of IFIs occurring within one month and 50% within one to three months after transplant. The proportion of non-albicans infections increased from 47% in the first month to 75% within the first three months (Table 8).

The invasive Saccharomyces infection was diagnosed on day 55, the invasive *Geotrichum capitatum* infection on day 3, *Mucor circinelloides* on day 77, and *Fusarium* spp. and *Penicillium* spp. each on day 26. The median time to diagnose within the group with prophylaxis was 14 days (3–55) and 13 days (7–43) in the group without.

### 3.7. Outcome

Eight patients (31%) with IFI died during the primary ICU stay. The death cause was in up to 88% of cases attributable to IFI (non-albicans species 50%, Aspergillus 38%). All-cause 90-day mortality after transplantation was 8%, with an IFI-related mortality rate of 53%.

Within the first year, 16 (62%) patients died, with the majority experiencing IFI. One-year survival was markedly reduced in cases of IFI compared to patients without IFI (39% vs. 91%, *p* < 0.001). Moreover, survival was distinctly impaired for patients with non-albicans Candida compared to the Candida albicans species (33% vs. 89%, *p* < 0.001). The subgroup analysis of one-year mortality based on the targeted antifungal prophylaxis showed a trend towards higher mortality in IFI patients not receiving prophylaxis.

Patients with an IA died within 12 ± 12 days due to septicemia and multiple organ failure. The one patient with invasive *Geotrichum capitatum* infection died due to sepsis on the 178th day in the hospital.

Patients with IFI had a twice as long ICU stay (10 vs. 5 days), developed more often postoperative renal failure (73% vs. 40%), and had a higher rate of ICU readmissions (17 vs. 10). Bile leaks were a common complication in this group (46% vs. 11%), followed by the need for re-operation (77% vs. 29%), and early re-transplantation (4 vs. 2), Table 3. Neither the duration of ventilation nor the duration of surgery showed a significant difference between the two groups (ventilation: 28 (2–2160) vs. 22 (1–2160) hours, *p* = 0.053; surgery: 362 (188–614) vs. 355 (173–783) minutes, *p* = 0.814).

Finally, we identified seven cases of drug-related adverse events, all of them micafungin associated. None of them were considered serious or dose-limiting (in three cases gastrointestinal symptoms occurred, in four cases, mild elevation of bilirubin and transaminase levels). All adverse events were reversible upon cessation.

## 4. Discussion

Liver transplantation has become a routinely performed and effective treatment for end-stage liver diseases. However, due to complex immunosuppression, infections are becoming the main determinant of morbidity and mortality in the early postoperative period. Besides bacteria and viruses, fungal infections are getting in the focus of outcome determining pathogens. Therefore, in our study, we reviewed the early postoperative course of 224 first-time OLT recipients under targeted echinocandin prophylaxis and found a 90-day IFI incidence of 12%. Based on the prespecified risk factors, we correctly classified the vast majority of patients as the IFI high-risk group and 83% received echinocandin prophylaxis. However, 21% of patients still developed an IFI. Furthermore, all-cause 90-day mortality after transplantation was 8%, with an IFI-related mortality rate of 53%. None of the patients with invasive aspergillosis survived. Despite targeted echinocandin prophylaxis, there is still a notable risk for IFI.

### 4.1. Incidence of Invasive Fungal Infections

The Transplant Associated Infection Network (TRANSNET) reported a one-year cumulative incidence of 4.7% for IFI in liver transplant recipients, with a 12-month mortality of 41% for patients with IA and 36% for IC [44]. As already reported by Raghuram et al., the spectrum of pathogens in our population is essentially dominated by *Candida* (81%), followed by *Aspergillus* (19%) [45]. 

In our work, we found an early postoperative IFI incidence of 7% for the first 21 days and 12% for 90 days, which is in line with the most recent USA study but higher than other reported European results [44,46]. Winston et al. compared anidulafungin to fluconazole for 21 days and found an overall IFI incidence of 5% for anidulafungin and 8% for fluconazole [47]. However, the Liver Transplant European Study Into the Prevention of Fungal Infection (TENPIN) study reported an incidence of only 1.4% at the end of 21-day prophylaxis with micafungin (2/140) being non-inferior to standard care prophylaxis with fluconazole, L-AmB, or caspofungin with an incidence of 0.7% (1/137) [46].

Considering breakthrough infections, we also observed a higher rate of 17% (18/105) in the first 90 days, compared to the findings published by Fortun et al. In their study on 71 high-risk liver transplant recipients receiving caspofungin for ≥21 days, they reported a successful treatment outcome (the absence of breakthrough IFI during the first 100 days) in 89% of the cases [48].

Our findings could be explained by the high percentage (79%) of ECD organs, the high rate (3%) of donor-derived infections, and, as described by Winston et al., the frequent and often underdosed prior antifungal therapy [26]. Moreover, 44% of patients experienced acute kidney injury with the need for renal replacement therapy. This can be interpreted in the context of the surgical technique, as the cava replacement was performed in 97% of cases requiring complete clamping of the vena cava. Consequently, cardiac output would be decreased by the reduced venous backflow to the right heart, congesting the renal venous drainage and endangering kidney function [49]. Finally, we observed a high degree of variability between the years examined as the incidence ranged from 4% to 20%, which will be the subject of further investigations. 

### 4.2. Targeted Antimycotic Prophylaxis

The main goal of targeted antimycotic prophylaxis is a clinically feasible identification of high-risk patients. In our study, 85% of patients were correctly recognized as a high-risk population for an IFI and received targeted antimycotic prophylaxis. Thus, we could demonstrate that the proposed algorithm for the stratification of the IFI risk is effective and feasible in a clinical setting.

Of the 26 invasive infections, 23 occurred within the algorithm-defined high-risk group. A total of 112 patients in the algorithm-defined low-risk group did not develop IFI, a further 86 were incorrectly classified as high-risk, and 3 as low-risk. Given the sensitivity of 89% (95%CI, 69.9–97.6) and a negative predictive value of 97% (95% CI, 92.8–99.1), we conclude that the used algorithm is suitable for screening to reduce the risk of IFI occurrence. By additional clinical evaluation, two more patients with delayed developed IFI were correctly identified and received antimycotic prophylaxis. Thus, if the algorithm had been implemented with even higher adherence, only one patient with IFI would not have received antimycotic prophylaxis. Therefore, we conclude that the echinocandin prophylaxis in our center was carried out in a very targeted manner. 

### 4.3. Time to Diagnosis

Most IFIs occur within two months following OLT [25,50,51,52], which is confirmed by our findings. Moreover, we draw the conclusion that more than half of the IFIs could already be diagnosed within the first two weeks after the operation. This might be due to the raised awareness of IFIs’ importance and the regularly performed microbiological screening during the ICU stay. Consistent with the data of Husain et al. and Singh et al., we also found a varying time course depending on the pathogen, with a median onset time of 13 days for IC and 36 days for IA [27,53,54]. Moreover, these authors describe all early IFIs occurring within the first month after OLT in more than two-thirds of the patients. These findings underline the importance of IFI screening in the early postoperative phase.

### 4.4. Composition and Sites of Pathogens

The main IFI sites in our population were blood-borne candidemia (48%) and intraabdominal candidiasis (67%), with isolated candidemia being in 100% of cases associated with indwelling catheters. Therefore, we could confirm the available evidence on the main infection sites after OLT [45,55,56]. Like in earlier reports, biopsy-proven *Candida esophagitis* was found in one case [27]. Moreover, Singh et al. noted that liver transplant recipients with IA were uniquely predisposed to dissemination beyond the lungs, with pulmonary aspergillosis being in only 31% and two or more non-contiguous organ sites in 58% involved. In the case of dissemination, the CNS was involved in 80% of the patients; other sites of involvement included the eye, heart, thyroid, pancreas, kidneys, urinary bladder, and thoracic spine [53]. In our study, none of the patients with IA showed an extrapulmonary involvement, most probably attributable to the fact that all patients died within an average of 12 days. Interestingly, 80% of the cases with IA showed a co-infection with a non-albicans Candida species or a non-Aspergillus mold infection (*Fusarium* spp., *Mucor* spp., and *Penicillium* spp.). 

In contrast to the constant distribution of the mold species, with the predominance of *Aspergillus fumigatus* over the years, a persistent rise of non-albicans *Candida* species has been recently seen among IC infections [27,53]. While a proportion of non-albicans *Candida* was described, with 35% in 2003 (*C. glabrata* 21%, *C. tropicalis* 9%, *C. parapsilosis* 3%, *C. guilliermondii* 3%), it already increased to 68% in 2012, having a tremendous impact on the one-year survival in the case of *C. parapsilosis* (28%), other non-albicans *Candida* (50%), *C. albicans* (75%), *Cryptococcus* (50%) and *Aspergillus* (50%). In addition to antibiotic spontaneous bacterial peritonitis prophylaxis before transplantation (especially the use of Fluoroquinolones), the relation of disease severity and the degree of underlying illness, the escalating use of azoles was further identified as an independent risk factor for increasing fungal colonization and subsequent non-albicans infections [27,45].

These findings are in line with our results, describing a 57% proportion of non-albicans species among the IFI patients with a likewise predominance of *C. glabrata* (33%) but a prominent frequency of *C. dubliensis* (33%). We identified only one patient with IC caused by *C. parapsilosis*, which was diagnosed three weeks after the OLT. This patient died due to fungal sepsis within 17 days after the diagnosis. We could confirm previous findings on a significantly reduced one-year survival in the case of non-albicans infections [45]. 

However, this finding should be interpreted critically, considering the use of selective digestive decontamination, the prolonged use of perioperative broad-spectrum antibiotics, and echinocandins in antifungal prophylaxis. Critically ill patients, the recipients of liver transplants, are more likely to be exposed to antibiotics, which not only act against potentially pathogenic microorganisms but also suppress the normal native flora of the gut and promote yeast overgrowth in the throat and gut. In the perioperative course of liver transplantation, medical procedures such as endotracheal intubation, intestinal manipulation, or the transient absence of enteral feeding and reduction of intestinal blood flow may lead to migration and translocation of the pathogens, causing pneumonia and/or septicemia. The use of selective decontamination of the digestive tract aims to maintain healthy anaerobic flora while neutralizing yeast overgrowth. Due to the low event rate of IFI, the clinical effectiveness of selective digestive decontamination in reducing systemic Candida infections remain unknown. Several trials showed an effective reduction of yeast-related outcomes (except for candidemia) at the price of more gram-positive cocci infections, with an immediate re-colonization after the decontamination was discontinued [57,58,59]. In this context, it remains unclear whether selective decontamination possibly initiates the emergence of azole-resistant strains. The choice of perioperative antibiotic prophylaxis is in line with international guidelines, but its duration should be critically questioned since there is no evidence for a prolonged duration of more than 48 h [60,61].

This trend of increasing non-albicans Candida infections in the last decade, partly being less susceptible to triazoles, is especially important in terms of antimycotic prophylaxis [27,45,62,63,64,65]. Consequently, echinocandins are being increasingly used as an alternative to the current guideline-recommended fluconazole and liposomal amphotericin B [46,47,48,66]. Established as a first-line treatment for IC, they have demonstrated broad efficacy with low toxicity and few drug–drug interactions [40,46,67,68,69,70]. Echinocandins show no relevant interaction, neither with the P450 cytochrome nor the P-glycoprotein systems and therefore, do not influence the pharmacokinetics of tacrolimus or cyclosporine-A while being concomitantly administered [71,72,73,74,75,76]. In contrast to fluconazole, no adaptation of dosage is needed in the case of renal replacement therapy [77].

### 4.5. Risk Factors for Infection

In our work, we could validate only one-third of the previously defined risk factors for the IFI occurrence: (1) age of the recipient, (2) split-liver transplantation, (3) intraoperative blood transfusion of more than 40 PRBC, (4) donor-derived infection, and (5) posttransplant dialysis [18,24,25,26,27,28,78,79,80]. Moreover, we could confirm similar findings regarding a reduced impact of intraoperative factors, as already described by Husain et al. and Raghuram et al. [27,45].

### 4.6. Outcomes

Adverse events related to antifungal prophylaxis occurred in only seven cases (7%), all of them being mild and reversible upon prophylaxis cessation. None of these were considered serious or dose limiting. However, mentioned complications could be underrepresented due to the retrospective nature of the study.

A recent meta-analysis of six randomized controlled trials confirmed a reduction of overall fungal infections (both IFIs and superficial infections) as a result of antimycotic prophylaxis. Moreover, the attributable mortality was reduced; however, there was no reduction in the overall mortality [29,30,31,32,33,34]. The beneficial effect of prophylaxis in the literature was predominantly associated with the reduction of C. albicans infections and mortality.

Despite targeted echinocandin prophylaxis, there is still a notable risk for IFI. Although 90 of the 109 (83%) so-defined, high-risk recipients received echinocandin prophylaxis, 23 (21%) developed an IFI. The allegation of too-short treatment duration (median 9 days) can be countered by the fact that the infection occurred in 85% of the cases during the ongoing treatment. Only in three cases did the infection occur after prophylaxis. However, in these cases, the median duration of prophylaxis was 17 days, which is within the current recommendations [15,81].

Finally, we found an overall ICU mortality of 31% for the patients with IFI, with an attributable 90-day mortality of 53%. Moreover, within the first year after OLT, 16 patients died, and the majority of them suffered from IFI. This led to a markedly reduced survival in the case of IFI, compared to patients without IFI (*p* < 0.001). Furthermore, survival was distinctly impaired for patients with non-albicans Candida compared to Candida albicans species (33% vs. 89%). Invasive aspergillosis had a 100% mortality, in accordance with previously published data [19,20,21,22,23,44].

In addition, we could confirm the impact of IFIs on the ICU-related outcome (e.g., longer ICU stay, higher rate of postoperative renal failure or ICU-readmission), as well as postoperative surgical complications (e.g., bile leak, re-operation, early re-transplantation).

### 4.7. Limitations

When interpreting this study, several limitations should be kept in mind. Due to its retrospective nature, a selection bias cannot be excluded. Moreover, patients with previous re-transplantations are excluded from this work, despite the fact that re-transplantation is a known risk factor for IFI. This heterogenic group of patients, with multiple hospital admissions, was limited with often incomplete documentation in regard to their pre-treatments, which would make any analysis rather complex and could have a distorting effect on the results. Although this is one of the largest European retrospective studies on the incidence of IFI in OLT recipients under targeted echinocandin prophylaxis, we cannot rule out the potential effect of missing variables. Moreover, due to the relatively small number of OLT recipients with IFI, drawing any firm conclusions is rather complex. Further studies with larger patient samples, multicenter study designs, or prospective trials are needed to confirm our findings. The systematized data collection and analysis of the solid organ transplant registries may result in additional evidence on the incidence and risk factors for IFI after OLT.

Finally, it is complex to discriminate IFI-related mortality from the potential effect of the underlying disease and postoperative course on the mortality. However, IFI had at least an impact on the postoperative course in our patient population. Moreover, the cause of death of 53% of patients dying within the first three months could be associated with IFI.

## 5. Conclusions

Despite a high degree of safety, further research is needed to evaluate the prophylactic use of echinocandins against the emergence of breakthrough infections and fluconazole-resistant pathogens, especially considering the adverse effects of non-albicans Candida species. Adherence to the internal prophylaxis algorithms is of immense importance, bearing in mind the high IFI rates in case the algorithm is not followed.

## Figures and Tables

**Table 1:** Perioperative risk factors for invasive fungal infections.

Preoperative	Intraoperative	Postoperative
MELD Score > 30	High-urgency transplantation	Early re-transplantation within 90 days after transplant
Pretransplant serum creatinine > 2 mg/dL	Split-liver transplantation	Relaparotomy after transplant
Fungal colonization at baseline	Intraoperative transfusion of ≥ 40 units of cellular blood products, including platelets, PRBC, and autotransfusion	Post-transplant dialysis
Antiinfective pretreatment within 3 months before transplant	Transplantation time > 10 h	Biliary leak
	Roux-en-y choledochojejunostomy	Cytomegalovirus viremia
	Donor-Derived Infection	

MELD: Model for End-Stage Liver Disease Score, PRBC: packed red blood cells.

**Table 2:** Sociodemographic and clinical characteristics of included patients (*n* = 224).

Characteristics		All Patients (*n* = 224)	No IFI (*n* = 198)	IFI (*n* = 26)	*p*-Value	Missing Data (*n*/Total)
Age (years)		57.2 ± 11.1	58.0 ± 10.5	51.8 ± 13.9	0.038	0/224
Male sex		172 (76.8)	154 (77.8)	18 (69.2)	0.459	0/224
Weight (kg)		81.5 ± 16.4	81.7 ± 16.0	80.2 ± 19.8	0.680	0/224
Height (cm)		174.4 ± 8.5	174.3 ± 8.4	175.5 ± 8.9	0.480	0/224
Body mass index (kg/m^2^)		26.8 ± 5.0	26.9 ± 4.9	25.9 ± 5.7	0.343	0/224
SAPS III score		45.0 ± 8.5	44.9 ± 8.6	45.8 ± 8.3	0.635	7/224
MELD score		14 (6–40)	14 (6–40)	16 (6–40)	0.174	7/224
Charlson comorbidity index		4 (0–12)	4 (0–12)	4 (0–9)	0.672	2/224
**Underlying disease**					0.198	0/224
Alcoholic liver disease		56 (25.0)	50 (25.3)	6 (23.1)	1.000	0/224
Malignancy and other tumors		92 (41.1)	83 (41.9)	9 (34.6)	0.531	0/224
	Hepatocellular carcinoma	85 (92.4)	76 (91.6)	9 (100.0)	0.710	
	Cholangiocellular carcinoma	3 (3.3)	3 (3.6)	0 (0.0)	0.528	
	Neuroendocrine tumor	3 (3.3)	3 (3.6)	0 (0.0)	0.528	
	Polycystic liver disease	1 (1.1)	1 (1.2)	0 (0.0)	0.716	
Virus related		9 (4.0)	8 (4.0)	1 (3.8)	1.000	0/224
Non-alcoholic fatty liver disease		14 (6.3)	14 (7.1)	0 (0.0)	0.380	0/224
Budd-Chiari syndrome		6 (2.7)	6 (3.0)	0 (0.0)	1.000	0/224
Acute liver failure		10 (4.5)	7 (3.5)	3 (11.5)	0.096	0/224
Cholestatic		17 (7.6)	14 (7.1)	3 (11.5)	0.426	0/224
Autoimmune hepatitis		8 (3.6)	6 (3.0)	2 (7.7)	0.234	0/224
Metabolic Liver Disease		10 (4.5)	9 (4.5)	1 (3.8)	1.000	0/224
Other		2 (0.9)	1 (0.5)	1 (3.8)	0.219	0/224

Abbreviations: SAPS III: simplified acute physiology score III; MELD: model of end-stage liver disease.

**Table 3:** Potential risk factors for an invasive fungal infection (*n* = 224).

Risk Factors		All Patients (*n* = 224)	No IFI (*n* = 198)	IFI (*n* = 26)	*p*-Value	Missing Data (*n*/Total)
**Preoperative risk factors**						
MELD Score > 30		20 (8.9)	16 (8.1)	4 (15.4)	0.263	7/224
Fungal colonization at baseline		15 (6.7)	10 (5.1)	5 (19.2)	0.019	0/224
Anti-infective pretreatment		30 (13.4)	25 (12.6)	5 (19.2)	0.360	0/224
Pretransplant serum creatinine > 2 mg/dL		21 (9.4)	19 (9.6)	2 (7.7)	1.000	0/224
**Intraoperative risk factors**						
Choledochojejunostomy, any time		24 (10.7)	15 (7.6)	9 (34.6)	<0.001	1/224
	Choledochojejunostomy, primary	15 (6.7)	11 (5.6)	4 (15.4)	0.080	1/224
Transplantation time > 11 h		4 (1.8)	4 (2.0)	0 (0.0)	1.000	2/224
Intraoperative blood transfusion > 40 PRBC		2 (0.9)	1 (0.5)	1 (3.8)	0.220	2/224
Split liver transplantation		6 (2.7)	3 (1.5)	3 (11.5)	0.022	0/224
Donor-derived infection		7 (3.1)	2 (1.0)	5 (19.2)	<0.001	0/224
High-urgency transplantation		9 (4.0)	6 (3.0)	3 (11.5)	0.079	0/224
**Postoperative risk factors**						
Bile leak		34 (15.2)	22 (11.2)	12 (46.2)	<0.001	1/224
	Choledochojejunostomy, secondary	9 (4.0)	4 (2.0)	5 (19.2)	0.001	1/224
Relaparotomy, any reason		77 (34.4)	57 (28.8)	20 (76.9)	<0.001	1/224
	Relaparotomy, bile leak related	29 (12.9)	19 (9.6)	10 (38.5)	<0.001	1/224
	Relaparotomy, not bile leak related	48 (21.4)	38 (19.2)	10 (38.5)	0.039	1/224
Early re-transplantation		6 (2.7)	2 (1.0)	4 (15.4)	0.002	0/224
Posttransplant dialysis		99 (44.2)	80 (40.4)	19 (73.1)	0.003	0/224
CMV viremia		64 (28.6)	54 (27.3)	10 (38.5)	0.252	0/224

Abbreviations: IFI: invasive fungal infection; PRBC: packed red blood cells; MELD: model of end-stage liver disease; CMV: cytomegalovirus; RRT: renal replacement therapy.

**Table 4:** Risk factors for IFI within 90 days of liver transplantation: univariate and multivariate Cox regression analyses (*n* = 224).

Nondependent Variable		B-Coefficient	*p*-Value	HR	95% CI		Multivariate Analysis	
					Lower	Upper	HR (95% CI)	*p*-Value
Age (years)		−0.039	0.007	0.96	0.94	0.99	0.97 (0.94; 0.99)	0.027
Sex (male)		0.427	0.315	1.53	0.67	3.53		
Height (cm)		0.015	0.523	1.02	0.97	1.06		
Weight (kg)		−0.006	0.626	0.99	0.97	1.02		
Body mass index (kg/m^2^)		−0.042	0.319	0.96	0.88	1.04		
SAPS III score		0.015	0.556	1.02	0.97	1.07		
MELD score		0.036	0.110	1.04	0.99	1.08		
Charlson comorbidity index		0.046	0.580	1.05	0.89	1.23		
**Underlying disease malignancy and other tumors (reference category)**								
	Alcoholic liver disease	0.040	0.939	1.04	0.371	2.925		
	Virus related	0.041	0.969	1.04	0.132	8.224		
	Non-alcoholic fatty liver disease	-	-	-	-	-		
	Budd-Chiari syndrome	-	-	-	-	-		
	Acute liver failure	1.337	0.051	3.81	1.030	14.079		
	Cholestatic	0.593	0.374	1.81	0.490	6.683		
	Autoimmune hepatitis	0.961	0.219	2.61	0.565	12.104		
	Metabolic Liver Disease	0.137	0.897	1.15	0.145	9.051		
	Other	1.986	0.060	7.28	0.920	57.692		
**Preoperative risk factors**								
MELD Score > 30		0.767	0.158	2.15	0.74	6.25		
Fungal colonization at baseline		1.293	0.009	3.65	1.37	9.67	1.75 (0.61; 5.04)	0.300
Anti-infective pretreatment		0.519	0.297	1.68	0.63	4.46		
Pretransplant serum creatinine > 2 mg/dL		−0.223	0.762	0.80	0.19	3.39		
**Operative risk factors**								
Choledochojejunostomy, primary		0.983	0.071	2.67	0.92	7.76		
Transplantation time > 11 h		−3.022	0.678	0.05	0.00	77.12		
Intraoperative blood transfusion > 40 PRBC		2.211	0.031	9.12	1.22	68.19	24.08 (2.72; 213.01)	0.004
Split liver transplantation		1.779	0.004	5.93	1.77	19.81	5.18 (1.40; 19.16)	0.014
Donor-derived infection		2.657	<0.001	14.25	5.34	38.01	9.70 (3.24; 29.04)	<0.001
High-urgency transplantation		1.406	0.022	4.08	1.22	13.60	1.72 (0.43; 6.95)	0.448
**Postoperative risk factors**								
Bile leak		1.690	<0.001	5.42	2.50	11.73		
Relaparotomy, any reason		1.960	<0.001	7.10	2.85	17.69	4.62 (1.67; 12.77)	0.003
Early re-transplantation		1.156	<0.001	3.18	1.86	5.42		
Posttransplant dialysis		1.312	0.003	3.71	1.56	8.84	1.83 (0.69; 4.87)	0.224
CMV viremia		0.488	0.226	1.63	0.74	3.59		

Variables with an increased hazard ratio for IFI: recipient age, intraoperative blood transfusion >40 PRBC, donor-derived infection, and relaparotomy. Variables excluded from the model (multicollinearity): bile leak and early re-transplantation. Abbreviations: SAPS III, simplified acute physiology score III; MELD, Model for End-Stage Liver Disease Score; IFI, invasive fungal infections; PRBC, packed red blood cells; CMV, cytomegalovirus; CI, confidence intervals; HR, hazard ratio.

**Table 5:** Transplantations and invasive fungal infections per year.

Year	Transplantations (*n* = 224)	IFI% (95% CI)
2017	54	18.5 (10.4–30.8)
2018	70	4.3 (1.5–11.9)
2019	46	4.4 (1.2–14.5)
2020	54	20.4 (11.8–32.9)

Abbreviations: IFI: invasive fungal infections; CI: confidence intervals.

**Table 6:** Patients with Invasive Candidiasis.

Entity of Invasive Candidiasis		Number of Patients (%)	Comment
Proven infections		15 (71)	
	Candidemia without deep-seated candidiasis	3 (14)	All catheter-associated
	Candidemia with deep-seated candidiasis	5 (24)	
	Deep-seated candidiasis without candidemia	7 (33)	
Probable infections			Recovery of *Candida* spp. in an intra-abdominal specimen obtained surgically or within 24 h from external drainage
	Deep-seated candidiasis without candidemia	6 (29)	

**Table 7:** Description of patients with invasive aspergillosis.

	Diagnostic Criteria								
Patient	Histopathologic Examination	Clinical Symptoms	Radiological Abnormalities	Culture	Galactomannan in BALF	PCR in Blood and BALF	Fungal Co-Infection	Time to Diagnosis (Day)	Time of Death (Day)
1	●	●	●				*Candida dubliensis*	43	57
2		●	●	●	●		Candida orthopsilosis *, Candida krusei *, Mucor circinelloides	78	110
3		●	●	●			*Candida glabrata*	36	43
4	●	●	●	●		●	*Fusarium* spp., *Penicillium* spp.	26	29
5	●	●	●	●	●	●	-	9	15

* Possible infection. Abbreviations: BALF: bronchoalveolar lavage fluid; PCR: polymerase chain reaction.

**Table 8:** Time to diagnosis for invasive fungal infections.

	Time from Transplantation		
Pathogen	Less than 14 Days (*n* = 14)	15–30 Days (*n* = 4)	31–90 Days (*n* = 8)
*Candida* spp.	6 (43)	2 (50)	1 (13)
Non-albicans	6 (43)	1 (25)	3 (38)
*Aspergillus fumigatus*	1 (7)	1 (25)	3 (38)
Other	1 (7)	-	1 (13)

## Data Availability

The datasets used and analyzed during the current study can be made available from the corresponding author upon reasonable request.

## Supplementary Materials

The following supporting information can be downloaded at: https://www.mdpi.com/article/10.3390/jcm12041520/s1, Table S1. STROBE Statement—Checklist of items that should be included in reports of cohort studies, Table S2. Comparison of patients with and without targeted antimycotic prophylaxis (*n* = 224).
