# Supplementary material for: Incidence of Invasive Fungal Infections in Liver Transplant Recipients under Targeted Echinocandin Prophylaxis
Source: J Clin Med. 2023 Feb 14;12(4):1520. doi: 10.3390/jcm12041520 (PMC9960065; doi:10.3390/jcm12041520)
Supplement: Supplementary file 1 [file jcm-12-01520-s001.zip › jcm-2190546-supplementary.pdf]

Article

Incidence of Invasive Fungal Infections in Liver Transplant Recipients under Targeted Echinocandin Prophylaxis

Robert Breitkopf, Benedikt Treml, Katharina Simmet, Zoran Bukumirić, Margot Fodor, Thomas Senoner and Sasa Rajsic

Supplementary Material

Contents

Table S1. STROBE Statement - Checklist of items that should be included in reports of cohort studies.
Table S2. Comparison of patients with and without targeted antimycotic prophylaxis (n = 224).

Table S1. STROBE Statement - Checklist of items that should be included in reports of cohort studies.

No.	Item	Recommendation	Page
	**Title and abstract**		
1		(*a*) Indicate the study's design with a commonly used term in the title or the abstract	1
		(*b*) Provide in the abstract an informative and balanced summary of what was done and what was found	1
	**Introduction**		
2	Background/rationale	Explain the scientific background and rationale for the investigation being reported	2
3	Objectives	State specific objectives, including any prespecified hypotheses	2, 5
	**Methods**		
4	Study design	Present key elements of study design early in the paper	2
5	Setting	Describe the setting, locations, and relevant dates, including periods of recruitment, exposure, follow-up, and data collection	2
6	Participants	(*a*) Give the eligibility criteria, and the sources and methods of selection of participants. Describe methods of follow-up	2-5
		(*b*) For matched studies, give matching criteria and number of exposed and unexposed	
7	Variables	Clearly define all outcomes, exposures, predictors, potential confounders, and effect modifiers. Give diagnostic criteria, if applicable	2-5
8*	Data sources/ measurement	For each variable of interest, give sources of data and details of methods of assessment (measurement). Describe comparability of assessment methods if there is more than one group	3-5
9	Bias	Describe any efforts to address potential sources of bias	3-5
10	Study size	Explain how the study size was arrived at	NA
11	Quantitative variables	Explain how quantitative variables were handled in the analyses. If applicable, describe which groupings were chosen and why	3-6
12	Statistical methods	(*a*) Describe all statistical methods, including those used to control for confounding	5-6
		(*b*) Describe any methods used to examine subgroups and interactions	5-6
		(*c*) Explain how missing data were addressed	
		(*d*) If applicable, explain how loss to follow-up was addressed	NA
		(*e*) Describe any sensitivity analyses	NA
	**Results**		
13*	Participants	(a) Report numbers of individuals at each stage of study—e.g. numbers potentially eligible, examined for eligibility, confirmed eligible, included in the study, completing follow-up, and analysed	5
		(b) Give reasons for non-participation at each stage	Fig. 1
		(c) Consider use of a flow diagram	Fig. 1
14*	Descriptive data	(a) Give characteristics of study participants (e.g., demographic, clinical, social) and information on exposures and potential confounders	Table 2
		(b) Indicate number of participants with missing data for each variable of interest	
		(c) Summarise follow-up time (e.g., average and total amount)	Tables
15*	Outcome data	Report numbers of outcome events or summary measures over time	Tables
16	Main results	(*a*) Give unadjusted estimates and, if applicable, confounder-adjusted estimates and their precision (e.g., 95% confidence interval). Make clear which confounders were adjusted for and why they were included	Tables
		(*b*) Report category boundaries when continuous variables were categorized	Tables
		(*c*) If relevant, consider translating estimates of relative risk into absolute risk for a meaningful time period	NA
17	Other analyses	Report other analyses done—e.g., analyses of subgroups and interactions, and sensitivity analyses	5-6
	**Discussion**		
18	Key results	Summarise key results with reference to study objectives	13

19	Limitations	Discuss limitations of the study, taking into account sources of potential bias or imprecision. Discuss both direction and magnitude of any potential bias	16
20	Interpretation	Give a cautious overall interpretation of results considering objectives, limitations, multiplicity of analyses, results from similar studies, and other relevant evidence	13-16
21	Generalisability	Discuss the generalisability (external validity) of the study results	14-16
	**Other information**		
22	Funding	Give the source of funding and the role of the funders for the present study and, if applicable, for the original study on which the present article is based	17

*Give information separately for exposed and unexposed groups.

Table S2. Comparison of patients with and without targeted antimycotic prophylaxis (n = 224).

Characteristics	NO TAP (*n* = 119)	TAP (*n* = 105)	OR	95% CI lower	95% CI upper	Missing Data (*n*/Total)
Age (years)	58.6 ±10.1	55.7 ±11.7	0.98	0.95	1.00	0 / 224
Male sex	94 (79.0)	78 (74.3)	1.30	0.70	2.42	0 / 224
Weight (kg)	83.3 ±15.0	79.5 ±17.9	0.99	0.97	1.00	0 / 224
Height (cm)	174.8 ±8.2	174.0 ±8.8	0.99	0.96	1.02	0 / 224
Body mass index (kg/m2)	27.3 ±4.5	26.2 ±5.4	0.96	0.91	1.01	0 / 224
SAPS III score	44.5 ±8.8	45.5 ±8.2	1.01	0.98	1.05	7 / 224
MELD score	12 (6-34)	15 (6-40)	1.09	1.04	1.13	7 / 224
Charlson comorbidity index	4 (0-12)	4 (0-10)	1.02	0.92	1.14	2 / 224
**Underlying disease: malignancy and other tumors (reference category)**						0 / 224
Alcoholic liver disease	30 (25.2)	26 (24.8)	1.41	0.7	2.77	
Virus related	4 (3.4)	5 (4.8)	2.04	0.51	8.10	
Non-alcoholic fatty liver disease	9 (7.6)	5 (4.8)	0.91	0.28	2.92	
Budd-Chiari syndrome	5 (4.2)	1 (1.0)	0.33	0.04	2.90	
Acute liver failure	0 (0.0)	10 (9.5)	-	-	-	
Cholestatic	5 (4.2)	12 (11.4)	3.91	1.27	12.04	
Autoimmune hepatitis	4 (3.4)	4 (3.8)	1.63	0.38	6.93	
Metabolic Liver Disease	5 (4.2)	5 (4.8)	1.63	.44	6.03	
Other	0 (0.0)	2 (1.9)	-	-	-	
**Preoperative risk factors**						
MELD Score >30	2 (1.7)	18 (17.1)	12.10	2.74	53.54	7/224
Fungal colonization at baseline	1 (0.8)	14 (13.3)	18.15	2.34	140.61	0/224
Antiinfective pretreatment	1 (0.8)	29 (27.6)	45.03	6.01	337.46	45.026
Pretransplant serum creatinine >2 mg/dl	6 (5.0)	15 (14.3)	3.14	1.17	8.42	0/224
**Operative risk factors**						
Choledochojejunostomy, any time	5 (4.2)	19 (18.1)	5.04	1.81	14.03	1/224
Choledochojejunostomy, primary	5 (4.2)	10 (9.5)	2.40	0.79	7.26	1/224
Transplantation time >11 hours	2 (1.7)	2 (1.9)	1.13	0.16	8.14	2/224
Intraoperative blood transfusion >40 PRBC	0 (0.0)	2 (1.9)	-	-	-	2/224
Split liver transplantation	0 (0.0)	6 (5.7)	-	-	-	0/224
Donor derived infection	0 (0.0)	7 (6.7)	-	-	-	0/224
High-urgency transplantation	0 (0.0)	9 (8.6)	-	-	-	0/224
**Postoperative risk factors**						
Bile leak (all)	3 (2.5)	31 (29.5)	16.06	4.74	54.42	1/224
Relaparotomy, any reason	17 (14.3)	60 (57.1)	8.00	4.21	15.21	1/224
Relaparotomy, bile leak related	0 (0.0)	29 (27.6)	-	-	-	1/224
Relaparotomy, not bile leak related	17 (4.3)	31 (29.5)	2.51	1.30	4.88	1/224
Posttransplant dialysis	33 (27.7)	66 (62.9)	4.41	2.51	7.75	0/224
CMV viremia	25 (21.0)	39 (37.1)	2.22	1.23	4.02	0/224

Abbreviations: IFI: invasive fungal infection; PRBC: packed red blood cells; MELD: model of end stage liver disease; CMV: cytomegalovirus; RRT: renal replacement therapy; OR: odds ratio, CI: confidence intervals.
